# The ethics of ‘Trials within Cohorts’ (TwiCs): 2nd international symposium: London, UK. 7-8 November 2016

**DOI:** 10.1186/s13063-017-1961-0

**Published:** 2017-07-20

**Authors:** Clare Relton, Maarten Burbach, Clive Collett, James Flory, Sophie Gerlich, Soren Holm, Amanda Hunn, Scott Y. Kim, Linda Kwakkenbos, Anne May, Jon Nicholl, Danny Young-Afat, Shaun Treweek, Rudolf Uher, Tjeerd van Staa, Joanne van der Velden, Helena Verkooijen, Andrew Vickers, Sophie Welch, Merrick Zwarenstein, Merrick Zwarenstein, Scott Kim, James Flory, Zachary Goodman, Søren Holm, Shaun Treweek, Sophie Gerlich, Anne M. May, Danny A. Young-Afat, Johannes P. Burbach, Carla H. van Gils, Rieke van der Graaf, Helena M. Verkooijen, Laura C. Coates, William Tillett, David Torgerson, Neil McHugh, Peter Taylor, Lesley Brown, Anne Heaven, John Young, Andrew Clegg, Kate Chatfield, Rudolf Uher, Anne M. May, Roxanne Gal, Evelyn M. Monninkhof, Danny A. Young Afat, Carla H. van Gils, Rolf H. H. Groenwold, Helena M. Verkooijen, Andrew Vickers, Linda Kwakkenbos, Marie-Eve Carrier, Brett D. Thombs, the SPIN investigators, Joanne M. van der Velden, A. Sophie Gerlich, Jorrit-Jan Verlaan, Helena M. Verkooijen, Alice M. Couwenberg, Rolf H. H. Groenwold, Rieke van der Graaf, Johannes P. M. Burbach, Joanne M. van der Velden, Anne M. May, Helena M. Verkooijen, Emily Peckham, Suzanne Crossland, Tom Hughes, Alisha O’Connor, Imogen Sargent, Simon Gilbody

**Affiliations:** 1 ScHARR, University of Sheffield, Sheffield, UK; 2 University Medical Centre, Utrecht, Netherlands; 3 Healthy Research Authority (HRA), London, UK; 4 Memorial Sloan Kettering Cancer Center, New York, NY USA; 5 University of Manchester, Manchester, UK; 6 National Institute for Health (NIH), Bethesda, MD USA; 7 McGill University, Montreal, Canada; 8 University of Aberdeen, Aberdeen, UK; 9 Dalhousie University, Halifax, Canada; 10 Halocline Coaching & Consultancy, Abderdeen, UK; 11 Western University, London, Ontario Canada; 12 Schulich School of Medicine & Dentistry, Western University, London, Ontario N6A 3K Canada; 13 Department of Bioethics, National Institutes of Health, Bethesda, MD USA; 14 Cornell University (Weill Cornell Medical College), Ithaca, NY USA; 15 Centre for Social Ethics and Policy, School of Law, University of Manchester, Manchester, UK; 16 Centre for Medical Ethics, HELSAM, University of Oslo, Oslo, Norway; 17 Department of Health Science and Technology, Aalborg University, Aalborg, Denmark; 18 Health Services Research Unit, University of Aberdeen, Aberdeen, AB25 2ZD UK; 19 Department of Radiation Oncology, University Medical Center Utrecht, Utrecht, The Netherlands; 20 Department of Clinical Epidemiology, Julius Center for Health Sciences and Primary Care, University Medical Center Utrecht, Utrecht, The Netherlands; 21 Department of Medical Humanities, Julius Center for Health Sciences and Primary Care, University Medical Center Utrecht, Utrecht, The Netherlands; 22 Nuffield Department of Orthopaedics, Rheumatology and Musculoskeletal Sciences, University of Oxford, Oxford, UK; 23 Department of Rheumatology, Royal National Hospital for Rheumatic Diseases, Bath, UK; 24 York Trials Unit, University of York, York, UK; 25 Department of Pharmacy and Pharmacology, University of Bath, Bath, UK; 26 Bradford Institute for Health Research, Bradford Teaching Hospitals NHS Foundation Trust, Bradford, UK; 27 Academic Unit of Elderly Care and Rehabilitation, University of Leeds, Leeds, UK; 28 Centre for Professional Ethics, University of Central Lancashire, Preston, UK; 29 Department of Psychiatry, Dalhousie University, Halifax, Nova Scotia B3H 2E2 Canada; 30 Department of Clinical Epidemiology, Julius Center for Health Sciences and Primary Care, University Medical Center Utrecht, Utrecht, The Netherlands; 31 Imaging Division, University Medical Center Utrecht, Utrecht, The Netherlands; 32 Memorial Sloan Kettering Cancer Centre, New York, NY USA; 33 Department of Psychiatry, McGill University, Montréal, Québec Canada; 34 Lady Davis Institute for Medical Research, Jewish General Hospital, Montréal, Québec Canada; 35 Behavioural Science Institute, Clinical Psychology, Radboud University, Nijmegen, The Netherlands; 36 Department of Radiation Oncology, University Medical Center Utrecht, Utrecht, The Netherlands; 37 Department of Orthopaedic Surgery, University Medical Center Utrecht, Utrecht, The Netherlands; 38 Julius Center for Health Sciences and Primary Care, University Medical Center Utrecht, Utrecht, The Netherlands; 39 Department of Radiation-Oncology, University Medical Center Utrecht, Utrecht, 3508 GA The Netherlands; 40 Julius Center for Health Sciences and Primary Care, University Medical Center Utrecht, Utrecht, 3508 GA The Netherlands; 41 Imaging Division, University Medical Center Utrecht, Utrecht, 3508 GA The Netherlands; 42 Mental Health and Addiction Research Group, Department of Health Science, University of York, York, UK; 43 Leeds and York Partnership NHS Foundation Trust, Leeds, UK; 44 Kent and Medway NHS and Social Care Partnership Trust, Kent, UK

## I1 2^nd^ TwiCs symposium summary

### Clare Relton^1^, Maarten Burbach^2^, Clive Collett^3^, James Flory^4^, Sophie Gerlich^2^, Soren Holm^5^, Amanda Hunn^3^, Scott Y Kim^6^, Linda Kwakkenbos^7^, Anne May^2^, Jon Nicholl^1^, Danny Young-Afat^2^, Shaun Treweek^8^, Rudolf Uher^9^, Tjeerd van Staa^5^, Joanne van der Velden^2^, Helena Verkooijen^2^, Andrew Vickers^4^, Sophie Welch^10^, Merrick Zwarenstein^11^

#### ^1^ScHARR, University of Sheffield, Sheffield, UK; ^2^University Medical Centre, Utrecht, Netherlands; ^3^Healthy Research Authority (HRA), London, UK; ^4^Memorial Sloan Kettering Cancer Center, New York, NY, USA; ^5^University of Manchester, Manchester, UK; ^6^National Institute for Health (NIH), Bethesda, MD, USA; ^7^McGill University, Montreal, Canada; ^8^University of Aberdeen, Aberdeen, UK; ^9^Dalhousie University, Halifax, Canada; ^10^Halocline Coaching & Consultancy, Abderdeen, UK; ^11^Western University, London, Ontario, Canada

##### **Correspondence:** Clare Relton (c.relton@sheffield.ac.uk)

**Abstract**

On 7-8^th^ November 2016, 60 people with an interest in the ‘Trials within Cohorts’ (TwiCs) approach for randomised controlled trial design met in London. The purpose of this 2^nd^ TwiCs international symposium was to share perspectives and experiences on ethical aspects of the TwiCs design, discuss how TwiCs relate to the current ethical framework, provide a forum in which to discuss and debate ethical issues and identify future directions for conceptual and empirical research.

The symposium was supported by the Wellcome Trust and the NIHR CLAHRC Yorkshire and Humber and organised by members of the TwiCs network led by Clare Relton and attended by people from the UK, the Netherlands, Norway, Canada and USA. The two-day symposium enabled an international group to meet and share experiences of the TwiCs design (also known as the ‘cohort multiple RCT design’), and to discuss plans for future research. Over the two days, invited plenary talks were interspersed by discussions, posters and mini presentations from bioethicists, triallists and health research regulators.

Key findings of the symposium were: (1) It is possible to make a compelling case to ethics committees that TwiCs designs are appropriate and ethical; (2) The importance of wider considerations around the ethics of inefficient trial designs; and (3) some questions about the ethical requirements for content and timing of informed consent for a study using the TwiCs design need to be decided on a case-by-case basis.

**Main report**

On 7-8^th^ November 2016, 60 people with an interest in the ‘Trials within Cohorts’ (TwiCs) design met in London for the 2^nd^ TwiCs international symposium. The symposium was supported by the Wellcome Trust and NIHR CLAHRC Yorkshire and Humber and organised by members of the TwiCs network led by Clare Relton. As well as UK participants, people came from the Netherlands, Norway, Canada and USA. Over the two days, the invited plenary talks were interspersed by discussions, posters and mini presentations from bioethicists, triallists and health research regulators.

On the first day (7^th^ November, 2016), Jon Nicholl (University of Sheffield, UK) opened the meeting, welcoming all to the symposium. He described how the first international symposium in 2014 brought together triallists using the design for the first time, and led to this, the 2^nd^ symposium which aimed to provide a forum in which to discuss and debate ethical issues including how the TwiCs approach relates to the current ethical framework.

**What are TwiCs?**

**Fig. 1 (abstract I1).**
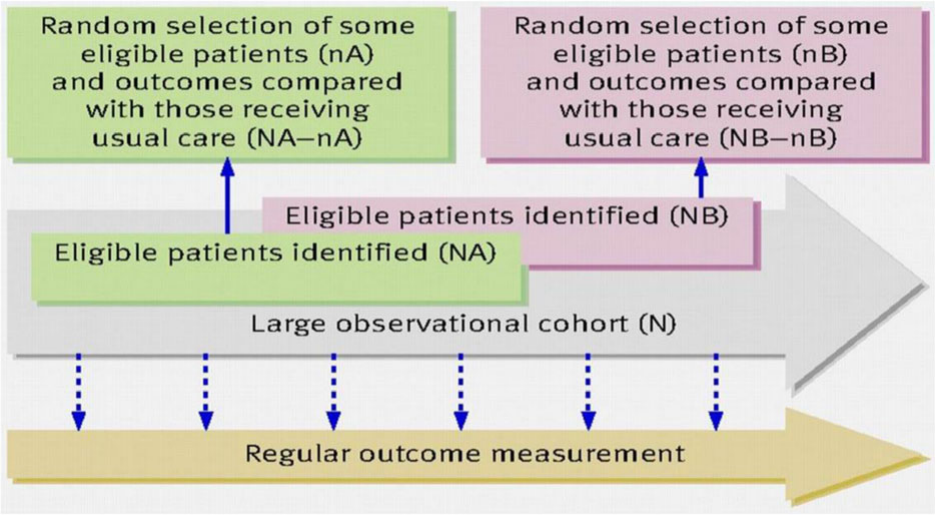
The ‘cohort multiple randomised controlled trial” design – BMJ

Clare Relton (University of Sheffield, UK) set the scene by outlining the Trials within Cohorts (TwiCs) approach as described in the original article (Fig. 1) in the BMJ in 2010 [1], and the 7 key features of the design:

(I) Recruitment of a large observational cohort of patients/ people with the condition of interest

(II) Regular measurement of outcomes for the whole cohort

(III) Capacity for multiple randomised controlled trials over time.

Then for each randomised controlled trial:

(IV) Identification of all eligible people in the cohort

(V) Random selection of some individuals from all eligible people in the cohort, who are then offered the trial intervention

(VI) Comparison of the outcomes in randomly selected people with the outcomes in eligible people not randomly selected; that is, those receiving usual care

(VII) “Patient centred” informed consent; that is, the process of obtaining information and consent aims to replicate that in routine health care as far as is possible.

**Ethics in current use**

Clare described how more than 20 studies using the TwiCs design now had ethics board approval from boards in Australia, Canada, Finland, France, Germany, Mexico, Netherlands, Spain, UK, and the USA. These studies were recruiting cohort populations (e.g. early life, children and adolescents, young indigenous, adults, older people) in a variety of settings (e.g., hospital, primary care), in order to facilitate trials in diverse health areas (e.g., attention deficit hyperactivity disorder, breast cancer, colo-rectal cancer, bone metastases, depression, hepatitis C, HIV, hip fracture, falls prevention, long term conditions, severe mental illness, scleroderma). Embedded within these cohorts, there were at least 20 randomised trials testing a wide range of interventions, and various approaches to informed consent were being used in these studies.

Jon Nicholl (University of Sheffield, UK) then gave an example of an emergency medicine research study where it was not possible to obtain informed consent prior to randomisation and have a viable trial, which illustrated that informed consent for participation in a trial is not always required. Jon argued that although all trial participants in TwiCs should receive information about data collection, storage and sharing and all other non-therapeutic research processes, only those in the intervention group need to receive information about the intervention. TwiCs designs randomly select from the cohort and offer the intervention being tested, those unselected are not actually **allocated** to ‘treatment as usual’, so there is no ethical obligation to tell those unselected about those who were selected, or about the treatment they are **not** being offered. Jon offered the analogy of lottery winners who are ‘selected’, where there is no sense in which ticket holders who don’t win are ‘allocated’ to a losers group. Jon concluded by offering a ‘Sheffield’ position statement for discussion *“In cohort trials, members of the cohort who are not selected to be offered a new treatment do not need to be told about the trial intervention (s)”.*

Merrick Zwarenstein (Western University, Ontario, Canada) set the context for the design by clarifying how pragmatic trials provide evidence to inform decision making and explanatory trials test whether or not an intervention causes an outcome. Merrick suggested that the PRagmatic Explanatory Continuum Indicator Summary -2 (PRECIS-2) framework could help designers of TwiCs trials match their design to their intentions. James Flory (Memorial Sloan Kettering Cancer Centre, New York, USA) outlined his review [2] of proposals for randomised controlled trials (RCTs) where randomisation occurred without prior information being given that interventions would be allocated at random (Randomisation without Consent). He described 6 different approaches found in the literature including emergency medicine research, Zelen designs and TwiCs designs.

The morning session concluded with two researchers reporting their practical experiences with the TwiCs design and the ethical questions that were generated and/or resolved through the use of the design. Rudolf Uher (Dalhousie University, Canada) described the FORBOW cohort of youth at high risk of severe mental illness and the first RCT (Skills for Wellness) embedded within this cohort. He described the advantages of using the design in a situation where most children at risk were not seeking help. No concerns had been raised about the TwiCs design during institutional review board process for FORBOW. Then Anne May (University Medical Centre, Utrecht, Netherlands) described the exercise-based FIT trial which is embedded within the hospital-based breast cancer ‘UMBRELLA’ cohort which uses the staged consent version of the TwiCs design [3]. She highlighted the possible pros (fast recruitment, no contamination) and cons (non-acceptance in the intervention group) using the TwiCs design.

**Ethical perspectives**

The afternoon session began with bioethicist Scott Kim (National Institute for Health, USA) who provided an overview of the ethical questions that pragmatic RCTs raise and an ethical analysis of two variations of TwiCs designs, those where information about (and consent for) future RCTs (i.e. the possibility of being randomised to the offer of a therapeutic intervention) was provided at enrolment to the cohort , and those where this information was only provided after randomisation to those in the intervention group. He concluded with system level ethical questions for broad population based TwiCs cohorts and learning healthcare systems. This was followed by Shaun Treweek (University of Aberdeen, Scotland) who focussed on the wider ethical question of the ethics of inefficiency, describing the lack of evidence to inform trial process decisions (e.g. ‘opt out’ vs ‘opt in’ for recruitment), and highlighting the potential waste of resources and participant goodwill. He argued that inefficiency is an ethical problem and how methodologists must generate evidence to support their decisions about trial processes. Tjeerd van Staa (University of Manchester, UK) described how TwiCs designs are suited for pragmatic trials in the era of big and ubiquitous data collection, but highlighted that refusal of treatment in the intervention arm could result in bias and loss of power. Andrew Vickers (Memorial Sloan Kettering Cancer Centre, USA) emphasised the benefits of integrating patient reported outcomes into routine clinical practice for optimizing clinical care, reusing these data for observational and experimental research such as TwiCs, and improving response rates.

Towards the end of the day, Kirsty Wydenbach from the MHRA (Medicines & Healthcare products Regulatory Authority) in the UK, emphasised that they were familiar with the TwiCs design and that their main concern was to ensure that participants in TwiCs were aware that they could withdraw at any time and that the requirements for safety monitoring were in place. Day one concluded with a speaker panel discussion on the ethics of whether or not to inform potential trial participants about interventions that they are not then subsequently offered if they are in the control (treatment as usual) group.

Day Two (November 8^th^, 2016) began with an overview of key findings of the previous day by Helena Verkooijen (University Medical Centre, Utrecht, Netherlands): including how a case can be made to ethics committees that TwiCs designs are appropriate and ethical; the importance of wider considerations around the ethics of inefficiency; and the range of perspectives on whether upfront information on randomisation to future interventions should be given at enrolment to the cohort. She summarised the discussion as ‘*If we don’t need to, why should we?’ Versus ‘And if we can do it, why shouldn’t we?’*

**Regulators perspective**

Clive Collett, Ethics Guidance & Strategy Manager at the UK Health Research Authority (HRA) argued that the methods and procedures used and the information provided should be proportionate to the nature and the complexity of the research, and the risks, burdens and potential benefits (to the participants and/or society) and the ethical issues at stake. He suggested that the closer the research is to standard clinical practice, the less need there is to provide patients and service users with detailed and lengthy information. The legal requirements for non-drug trials are that information must be provided regarding the broad nature and purpose of the research, the material and significant risks and benefits and alternatives, but that written evidence of consent was not legally required. He outlined forthcoming HRA guidance on applying a proportionate approach to the process of seeking consent which will allow the consent process to take place at the consultation using brief information sheets that promote genuine understanding.

Amanda Hunn, Joint Head of Policy and Public Affairs at the HRA sketched plans for a survey of Research Ethic Committee (REC) members in England to explore their appetite for five different study designs where there was randomisation without prior information being given that interventions would be allocated at random. Sophie Welch (Independent Ethics Consultant) emphasised the importance of dialogue with ethics boards, and suggested that researchers should not avoid the design on the assumption that it would not secure ethical approval.

Sophie then detailed 5 different questions that ethics committee members are likely to consider during ethical review: 1) does the proposed research respect the rights, autonomy, dignity, and wellbeing of the participant?, 2) is there a sound ethical basis for this research design?, 3) based on my experience, what do I think to this approach?, 4) what are the views of other committee members, and what guidance and/or regulation can we draw on?, and 5) have similar research designs already received ethical approval?

Bioethicist Søren Holm (University of Manchester, UK) explored why and when control groups should consent and whether ethical considerations relating to harm, burden, rights and reasonable expectations help us to answer this question. He concluded his talk with an exploration of what might be the reasonable expectations from the ordinary understanding of the patient-healthcare provider relationship.

**Engagement with ethics committees**

The morning session concluded with two researchers describing their experiences of using the TwiCs design and the ethical questions that were generated and/or resolved through use of the design. Linda Kwakkenbos (McGill University, Canada), reported that the Scleroderma Patient-centered Intervention Network (SPIN) Steering Committee and Patient Advisory Board liked that the TwiCs design was providing a sustainable framework for multiple trials of interventions for the rare disease scleroderma, and that the design did not engender disappointment for those patients not receiving intervention. Since the start of enrolment in the SPIN Cohort more than 1500 patients with the rare disease scleroderma had been enrolled from 39 centres in 5 countries after obtaining approval from the local ethics board for each centre.

Sophie Gerlich (University Medical Centre, Utrecht, Netherlands) discussed preliminary results of her study of patient understanding and opinions regarding informed consent with data drawn from questionnaires to patients who had either agreed or declined to participate in three cohorts using the TwiCs design – colorectal cancer, breast cancer and bone metastases.

After the lunchtime poster presentations, Danny Young-Afat (University Medical Centre, Utrecht) introduced the afternoon session which was devoted to 8 mini talks on future directions for empirical and conceptual research in relation to the TwiCs design. The session began with Joanne van der Velden (University Medical Centre, Utrecht) discussing the interim results regarding recruitment and randomisation for their ongoing Vertical RCT embedded in the PRESENT bone metastases cohort. She noted that these compared favourably to a classic multi-centre RCT in the same patient population which is running simultaneously in the Netherlands.

**Future research**

The remaining sessions explored future plans relating to the TwiCs design with a focus on ethical aspects. Petter Viksveen (University of Stavanger, Norway) outlined early plans to set up a mental health cohort for adolescents in Norway. Joanne Zakrewska (Pain Management Centre, UCLH NHS Foundation Trust, London, UK) argued for the need for a cohort study using the TwiCs design to facilitate the testing of surgical and pharmacological interventions for patients suffering from Trigeminal Neuralgia. Amanda Hunn (Health Research Authority (HRA), UK) described HRA plans to set up a special panel to give endorsement for registries that recruit patients into research (this includes ‘consent for consent’ and ‘consent to be approached’ registries such as the Yorkshire Health Study and Health Wise Wales). Panel endorsement of registries would mean that a study using an endorsed register/cohort to recruit would not require the ethics board to look at the recruitment process again as it would already have been endorsed by the HRA. Andrew Vickers (Memorial Sloan Kettering Cancer Center, USA) discussed the overzealous approach to autonomy of standard informed consent procedures and the harm which often arose from information overload for those patients randomised to usual care. He illustrated this with an example of a trial where late stage cancer patients make heart wrenching decisions about whether to risk possible side-effects for uncertain harms and then for 50% of the patients then randomised to usual care, this agonizing consent process makes no difference to their care and they could have been spared considerable anxiety and decisional burden. He argued for empirical research to document any consent-related distress and how this might be ameliorated by alternative approaches such as the TwiCs patient centred approach to informed consent.

The session concluded with three proposals for further research from Clare Relton (ScHARR, University of Sheffield). The first suggestion was to compare the efficiency and acceptability of two different Informed Consent pathways (Standard vs Tailored) for effectiveness trials with ‘usual care’ comparators. Efficiency would be measured using the ratios of numbers analysed, to the numbers: (i) approached, (ii) randomised, (iii) allocated to the intervention, (iv) accepting their allocation; as well as the representativeness of the population recruited, and the time taken and cost incurred. The second suggestion was to introduce an ‘Information and Consent’ extension to the CONSORT flow diagram and/or statement, and the third was to explore the potential of the TwiCs approach to transform healthcare systems into learning healthcare environments – linking up existing cohorts or even building a UK NHS based national cohort.

Day two concluded with a lively and wide ranging panel discussion with many contributions from the audience including the announcement that £1.1mn NIHR funding had just been obtained for a TwiCs designed study which was trialling a range of investigational medicinal products. The panel acknowledged that it was clearly possible to make a compelling case to ethics committees that TwiCs designs were appropriate and ethical; the importance of wider considerations around the ethics of inefficient trial designs; that there was broad consensus from those attending the symposium that there were no hard and fast rules regarding the informed consent processes relating to therapeutic processes (interventions, randomisation), and that some key ethical questions about the content and timing of informed consent for a TwiCs may need to be decided on a case-by-case basis.

The slides and films from the TwiCs Ethics symposium can be viewed at https://www.twics.global/ethics-symposium-2016

**Declaration**

**-Competing interests**

The authors have no competing interests

-**Funding**

The symposium was made possible by funding from the Wellcome Trust and NIHR CLAHRC Yorkshire and Humber. The publication costs of the meeting will be funded by the NIHR CLAHRC Yorkshire and Humber.

-**Authors’ contributions**

CR wrote the first draft and subsequent drafts with contributions from all authors.

**-Acknowledgments**

We would like to thank the funders, all the speakers and presenters and all those attended the conference.

**References**

1. Relton C, Torgerson D, O’Cathain, Nicholl J. Rethinking pragmatic randomised controlled trials: Introducing the ‘cohort multiple randomised controlled trial’ design. BMJ 2010; 340: c1066–c1066.

2. Flory JH, Mushlin AI, Goodman ZI. Proposals to Conduct Randomized Controlled Trials Without Informed Consent: a Narrative Review. Journal of general internal medicine. 2016 Dec 1;31(12):1511-8.

3. Young-Afat DA, Verkooijen HA, van Gils CH, van der Velden JM, Burbach JP, Elias SG, van Delden JJ, Relton C, van Vulpen M, van der Graaf R. Brief Report: Staged-informed Consent in the Cohort Multiple Randomized Controlled Trial Design. Epidemiology. 2016 May 1;27(3):389-92.

## ABSTRACTS

### Topic 1: Context

#### A1 TwiCs RCTs can be explanatory, pragmatic or in-between

##### Merrick Zwarenstein (merrick.zwarenstein@ices.on.ca)

###### Schulich School of Medicine & Dentistry, Western University, London, Ontario, N6A 3K, Canada

Randomised controlled trials (RCTs) using the TwiCs design streamline patient recruitment by tailoring and staging consent, allow for testing of multiple interventions against a common control group, and integrate evaluation into the natural flow of care. Are they pragmatic?

Schwartz and Lellouch [1] identified two opposite attitudes or purposes to RCT design: to provide evidence which supports a clinical, service delivery or health policy decision (the “pragmatic” attitude) or to explore a mechanism of action of the intervention under study (by testing whether or not it causes an outcome -the “explanatory” attitude).

These attitudes are not dichotomous, but represent opposite ends of a spectrum. The Pragmatic Explanatory Continuum Indicator Summary second generation tool (PRECIS-2) [2] operationalises the pragmatic/explanatory spectrum as 9 domains (Table 1), each reflecting an aspect of RCT design, each rated on a 5 point scale for similarity to usual care, as ordinarily provided in the clinical settings in which the intervention is intended to be used after evaluation in the planned RCT.

PRECIS-2 can be applied to all trials, including TwiCs trials, in order to help designers match the design of their trial to their intended use for the trial results. For example, the position on the PRECIS-2 scale for eligibility of a TwiCs RCT of treatments at a breast cancer clinic would be determined by how the control and intervention participants are selected from the entire cohort: more pragmatic if all clinic patients are included in the trial and if a representative subsample is selected for intervention, or more explanatory, if tight inclusion criteria exclude many clinic patients from the trial participants. This design choice can be repeated for the other eight PRECIS-2 domains.

**References**

1. Schwartz, D., Lellouch, J. (1967), ‘Explanatory and pragmatic attitudes in therapeutic trials’, Journal of Chronic Disease,20, 637-48.

2. PRECIS-2 https://www.precis-2.org/

**Table 1 (abstract A1).** Attitude of RCTs: Domains of PRECIS2

1. *Eligibility*: How similar are trial participants to those in intended usual care?
2. *Recruitment*: Is trial recruitment process similar to that of entry into usual care?
3. *Setting*: How similar is the trial setting to the intended usual care setting?
4. *Organisation*: Are resources added for care in the trial beyond those in usual care?
5. *Flexibility-delivery*: Are clinicians constrained differently from usual care?
6. *Flexibility-adherence*: Are participants selected, burdened beyond usual care?
7. *Follow up:* Are participants followed up more closely that in usual care?
8. *Primary outcome*: How similar are trial outcomes to usual care criteria for success?
9. *Primary analysis*: Are all participants included in the main analysis?

#### A2 Ethical Issues in Trials within Cohorts and other Pragmatic RCTs

##### Scott Kim (scott.kim@nih.gov)

###### Department of Bioethics, National Institutes of Health, Bethesda, MD, USA

The pragmatic imperative in clinical trials implies designing trials that mimic the day-to-day operations of the clinic, creating unique ethical questions that are shared by different types of pragmatic studies. In RCTs that compare two standard of care treatments, all patients inside the RCT receive ‘accepted standard treatments’ yet this does not imply there is minimal risk or that no consent is required [1]. In Trials within Cohorts (TwiCs), an RCT can be conducted in which the control groups receives just normal clinical care, without any experiences or changes to their care caused by the RCT. How does this difference affect the requirements of informed consent for TwiCs?

Intentionally withholding information about future randomisation and use of data—when it could be disclosed without detriment to the RCT—may run the risk of violating reasonable expectations of patients and compromising the trust that is so necessary for a stable program of pragmatic trials. This concern could be addressed in cohorts with TwiCs by disclosing information and obtaining permission from cohort participants ahead of time, in anticipation of future cohort-embedded RCTs. However, there is no widely accepted standard for the content of such prior disclosure and consent. Some investigators rely on non-TwiC specific general permission about ‘future use of data’ while others use pre-randomisation broad consent that includes explicit information about future randomised TwiCs (including information on randomisation, potential for future contact for the intervention arm, and use of data without further notification in control arm) [2]. For the time being, which of these options to use must be evaluated for each trial by considering the nature of the cohort, the interventions to be tested, and the reasonable expectations of patients in that context. However, the growing field of TwiCs may benefit from a more generalizable model of ethical analysis regarding the content and procedures of prior broad consent for TwiCs.

**References**

1. Chen SC, Kim SY. A framework for analysis of research risks and benefits to participants in standard of care pragmatic clinical trials. Clinical Trials. 2016;13(6):605-611.

2. Young-Afat DA, Verkooijen HM, Van Gils CH, et al. Staged-informed consent in the cohort multiple randomised controlled trial design. Epidemiology (Cambridge, Mass.). Jan 6 2016.

#### A3 Randomisation without Consent

##### James Flory, Zachary Goodman

###### Cornell University (Weill Cornell Medical College), Ithaca, NY, USA

####### **Correspondence:** James Flory (jaf9052@med.cornell.edu)

Informed consent to research is acknowledged to be a burdensome process for both researchers and participants. Various efforts have been made to improve it, including shorter consent forms, abbreviated oral consent, staged consents, and even omission of consent entirely. The last approach (‘randomization without consent’ or RWOC) is the most controversial.

Our review identified ten distinct designs for RWOC, falling into three general categories [1]. First are studies in which informed consent is not obtained because it is infeasible. The clearest example is emergency research, when extremely ill patients must be assigned treatment under rushed circumstances that preclude obtaining consent. Some forms of cluster randomized studies also fit this category.

A second category is studies in which consent is feasible, but arguably ethically unnecessary because the risk to participants from participation in a particular study is very low. A further argument is that consent requirements actually do harm in these cases, by making it more difficult to conduct very safe studies that stand to benefit patients by better informing clinical care. These proposals have been strongly criticized on the grounds that they deceive patients and disrespect patient autonomy [2]. Recent proposals of this type have focused on the possibility that they can be more ethically conducted in the context of a learning healthcare system [3].

A third category is known variously as ‘post-randomization consent’ or ‘Zelen’ designs. There are several versions of this design. But, the fundamental concept is that while patients who receive experimental interventions should give consent, patients who receive usual care as part of the control group may not need to consent. Indeed, in some cases asking patients in the control group for consent may create distress and disappointment without clear benefits to patients. Post randomization consent designs have generally attracted ethical scrutiny and criticism, and remain fairly rare in practice.

The TwiCs design (referred to in our original review as cohort multiple RCT [cmRCT]) is closely related to other post-randomization consent designs. A crucial design issue in a TwiCs is whether patients who are enrolled in a cohort give a broad consent that explicitly mentions future randomizations. If so, TwiCs is not RWOC, but a form of staged consent. The ethical arguments and intuitions as to whether a given implementation of TwiCs should mention randomization in the initial broad consent may vary significantly depending on the specific clinical question and context.

**References**

1. Flory JH, Mushlin AI, Goodman ZI. Proposals to Conduct Randomized Controlled Trials Without Informed Consent: a Narrative Review. J Gen Intern Med. 2016 Dec;31(12):1511-1518

2. Kim SY, Miller FG. Informed consent for pragmatic trials--the integrated consent model. N Engl J Med. 2014 Feb 20;370(8):769-72.

3. Faden RR, Beauchamp TL, Kass NE. Informed consent, comparative effectiveness, and learning health care. N Engl J Med. 2014;370(8):766–8.

#### A4 Why and when should control groups consent? Do ethical considerations relating to harm, burden, rights and reasonable expectations help us to answer this question?

##### Søren Holm^1,2,3^ (soren.holm@manchester.ac.uk)

###### ^1^Centre for Social Ethics and Policy, School of Law, University of Manchester, Manchester, UK; ^2^Centre for Medical Ethics, HELSAM, University of Oslo, Oslo, Norway; ^3^Department of Health Science and Technology, Aalborg University, Aalborg, Denmark

Informed consent in research ethics is a ‘technology’ designed to protect a number of basic ethical values. This talk outlined four sets of considerations that all lend support to a consent requirement (see below) and explored their implications for consent of control groups in Trials within Cohorts designs (TwiCs).

The most general justification for consent is that participants need to know that they are part of research and need to agree to this. But in standard clinical research, e.g. randomised controlled trials there are further reasons why we require consent from both the intervention and the control group. Control group participants in standard clinical research rarely receive ‘standard treatment’, i.e. the treatment they would have had if they were not research participants. Their treatment is often precisely specified and they are subject to additional diagnostic tests. This is one of the main reasons for seeking informed consent. Consent is needed if the research involves: 1) additional risk of harm, 2) additional burden, 3) infringement of rights relating to self-determination, privacy or bodily integrity, or 4) something that breaches the reasonable expectations of participants in relation to their contact with health care professionals.

A TwiCs trial can be designed so that there is no additional risk or burden because nothing changes for the control group; and all participants consent to the use of their data when entering the cohort. The need for informed consent from the control group can therefore, to a large extent be ‘designed out’, if the initial information to cohort participants explains the embedding of future trials in the cohort and no attempts are made to standardise the treatment of control group participants once they have been allocated to the control group.

#### A5 The ethics of inefficiency

##### Shaun Treweek (streweek@mac.com)

###### Health Services Research Unit, University of Aberdeen, Aberdeen, AB25 2ZD, UK

**Background**

Trials are important but they are sometimes inefficient. If inefficiency means that a trial has poor or irrelevant research questions, has made design choices that reduce relevance, has design inefficiencies that mean the trial cannot answer its research questions, or uses more resources than it needed to, then important ethical questions are raised.

**Discussion**

Broadly speaking, we can define two types of efficiency: scientific and process. Scientific efficiency is about choosing the right research questions and then the right design to answer those research questions. Scientific inefficiency can fatally wound a trial long before it comes into contact with a participant. For example, many trials are started in complete ignorance of earlier trials or systematic reviews that are highly relevant to the design decisions of the new trial, including whether the new trial is needed at all. This is a major ethical failure. Poor choice of outcomes or participants can render the results of an otherwise well-done trial irrelevant to the decisions it was intended to support.

Process efficiency is about proper planning and doing what you need to do to answer the research questions, and no more. Lack of evidence to inform trial decisions means that remarkably few trial process decisions can be described as evidence-informed. This process inefficiency wastes resources, adds burden to health research infrastructure and spills participant goodwill, again raising ethical questions. The TwiCs approach to pragmatic trial design is potentially more efficient than the standard approach pragmatic RCT design in a number of ways. For example, embedding trials within large observational cohorts can facilitate (i) fast and efficient recruitment of participants, (ii) collection of long term outcomes and (iii) enables the use of unequal randomisation (improving the efficiency of trials of high cost interventions compared with equal allocation).

There can be a tension between the ethics of making a design or process choice and the ethics of not making that same choice (opt in versus opt out for recruitment is an example, or the use of telephone reminders to non-responders). Ethical judgements need to consider this balance, not just the ethics of the initiative being proposed.

**Conclusion**

Inefficiency in research is an ethical problem. Trialists need to identify existing research before starting their trial design, and think carefully about who their trial is for so as to avoid irrelevance. Ethics and other governance structures need to ensure that this is done before granting approvals. Methodologists need to generate more evidence to support trial design and process decisions. Initiatives such as Trial Forge (http://www.trialforge.org) and the REWARD Alliance (http://rewardalliance.net) have important roles to play in this endeavour.

### Topic 2: Issues

#### A6 What do patients understand of the cohort multiple randomised controlled trial (cmRCT) design?

##### Sophie Gerlich^1^, Anne M. May^2^, Danny A. Young-Afat^1,2^, Johannes P. Burbach^1^, Carla H. van Gils^2^, Rieke van der Graaf^3^, Helena M. Verkooijen^1^

###### ^1^Department of Radiation Oncology, University Medical Center Utrecht, Utrecht, The Netherlands; ^2^Department of Clinical Epidemiology, Julius Center for Health Sciences and Primary Care, University Medical Center Utrecht, Utrecht, The Netherlands; ^3^Department of Medical Humanities, Julius Center for Health Sciences and Primary Care, University Medical Center Utrecht, Utrecht, The Netherlands

####### **Correspondence:** Danny A. Young-Afat (D.A.YoungAfat@umcutrecht.nl)

**Background**

Some ethical concerns have been raised following the introduction of the cmRCT design. This abstract addresses patients’ perspectives and understanding of the cmRCT design.

**Method**

At the University Medical Center Utrecht, patients with colorectal cancer, breast cancer and bone metastases are recruited into cohorts with a cmRCT design. In these cohorts, several trials are running. Self-administered questionnaires were used to evaluate patients’ opinion and understanding of the cmRCT design at several stages of cohort participation, i.e. shortly after agreeing to (n = 312) or declining (n = 84) cohort participation, after enrolment in a cohort based trial (n = 35), after declining participation in a trial (n = 20) and one to eight months after cohort enrolment (n = 56).

**Results**

This quantitative study is based on 507 returned questionnaires. Almost all patients (94%) indicated altruism as the main reason for cohort participation. Shortly after enrolment 5% (14/304) of patients did not remember whether they had agreed to future randomisation and 50 patients (16%) recalled this decision incorrectly. One to eight months after enrolment, 29% (16/56) did not remember agreeing to future randomisation, and 30% who thought not to have given permission for randomisation actually did. Shortly after enrolment, 35% (75/217) of patients who gave permission for randomisation hoped to receive an invitation for an experimental intervention. Forty-two percent (23/55) of patients who were offered an experimental treatment understood that selection for experimental interventions was based on chance, whereas 46% (25/55) of patients were not interested in understanding the selection procedure.

**Conclusion**

Altruism was the most important motivation for patients to participate in the cohorts. Some misconceptions exist regarding components of informed consent. However, these misconceptions do not seem to be more prevalent in cmRCT than in standard RCTs.

**Acknowledgements**

The abstract is being presented on behalf of the cmRCT study group. We thank the study team of the PLCRC, PRESENT and UMBRELLA cohort at the department of Radiation Oncology, Imaging Division and Julius Center of the University Medical Center Utrecht (UMCU) for running the cohort and collecting the data.

#### A7 Ethical Issues in a TwiCs study in early PsA

##### Laura C Coates^1^, William Tillett^2^, David Torgerson^3^, Neil McHugh^2,4^, Peter Taylor^1^

###### ^1^Nuffield Department of Orthopaedics, Rheumatology and Musculoskeletal Sciences, University of Oxford, Oxford, UK; ^2^Department of Rheumatology, Royal National Hospital for Rheumatic Diseases, Bath, UK; ^3^York Trials Unit, University of York, York, UK; ^4^Department of Pharmacy and Pharmacology, University of Bath, Bath, UK

####### **Correspondence:** Laura C Coates (Laura.coates@ndorms.ox.ac.uk)

**Study Design**

This study will recruit newly diagnosed psoriatic arthritis (PsA) patients. The cohort will receive best practice therapy following European Recommendations: a “treat to target” approach where treatment is escalated aiming for an objective target. Treatment will be escalated from one standard disease-modifying agent (DMARD), combination DMARDs and finally to biologics.

Two initial studies are planned. A randomised feasibility study will assess whether patients with mild PsA could be treated conservatively without DMARDs. A powered trial in moderate/severe PsA with two interventional arms will test more intensive drug therapies within the treat to target approach. One arm will receive combination DMARDs from the time of diagnosis, the other arm will receive an initial 6 month course of biologics tapering to DMARDs after this.

**Ethical issues raised by the TwiCs design**

The TwiCs design was chosen to allow analysis of real life outcomes in the cohort and treatment comparisons in the trials, producing generalizable results with the aim of changing routine practice.

**Positive ethical issues**

It will not be practical to “blind” therapy in these studies. This raises the issue of disappointment bias in patients who receive the “treatment as usual” comparator if they are aware that they have not been given the more intensive treatment. The use of the TwiCs design will avoid any disappointment bias allowing accurate comparisons of treatment.

**Ethical issues of concern**

The two stage consent for the cohort and then potentially for an interventional study must occur prior to starting treatment in newly diagnosed patients. Appropriate assessment at baseline in the cohort will have to rapidly allow randomisation to interventions and the consent forms and information given will have to be easily understandable to the patients to avoid overwhelming them.

In a usual RCT design, only patients consenting to the interventional study would be included in an intention to treat analysis. Whilst we plan to use complex statistical methods to adjust for the patients offered the intervention but declining (based on CACE analysis), a low consent rate for the offered interventions may affect our later analysis.

The studies planned within the TwiCs are both controlled trials of investigational medicinal products. As patients within the cohort will be acting as a “control” group for the interventions, additional detailed information relating to adverse events will have to be collected in the cohort beyond that which would normally be collected in a cohort placing an additional burden on these patients.

#### A8 The importance of oversight in Trials within Cohorts (TwiCs): experiences from the Community Ageing Research 75+ (CARE 75+) study

##### Lesley Brown^1^, Anne Heaven^1^, John Young^2^, Andrew Clegg^2^

###### ^1^Bradford Institute for Health Research, Bradford Teaching Hospitals NHS Foundation Trust, Bradford, UK; ^2^Academic Unit of Elderly Care and Rehabilitation, University of Leeds, Leeds, UK

####### **Correspondence:** Lesley Brown (Lesley.brown@bthft.nhs.uk)

The Community Ageing Research 75+ (CARE 75+) study is using a Trials within Cohorts (TwiCs) design to build ageing research capacity. TwiCs is also considered a useful method for recruiting potentially hard to reach groups such as people with frailty1. As part of the NIHR Collaboration for Leadership in Applied Health Research and Care Yorkshire & Humber (NIHR CLAHRC Y&H), CARE 75+ is recruiting community-dwelling older people (≥75 years) from across the UK (n =280) . The primary objective is a prospective epidemiological analysis of frailty, disability and quality of life trajectories with health, social and economic data captured at baseline, 6, 12, 24 and 48 months.

There are recognised challenges of using a multiple studies platform which have relevance for other researchers studying frailty, disability and cognitive impairment. We have observed that the initial cohort consent procedure is more complex and time-consuming than usual as researchers explain about requests for future study participation and that their data may be used as control data. We have also observed confusion between involvement the CARE 75+ observational study and involvement in sub-studies with participants sometimes not recognising the discrete nature of different studies.

To meet these challenges we have worked alongside our theme-wide patient and public group, the Frailty Oversight Group (FOG)2. Firstly, the FOG have scrutinised all sub-studies at the outset to ensure there is no unnecessary burden for participants, such as replication of outcome measures. Secondly, the FOG have observed researchers undertaking consent and assessments to improve the process from the participants’ perspective. Thirdly, we inform all participants of new up-and-coming studies by newsletters but stagger invitations to ensure equal access whilst not overburdening them with information. We will continue to work with the FOG to assist with improving branding of information sheets and consent forms to ensure clarity and discrimination between CARE 75+ and sub-studies. To date, 83% of CARE 75+ participants agreed to contact about future studies (e.g. qualitative research and pilot work for future randomised controlled trials).

**References**

1. Clegg A, Relton C, Young J, Witham M. Improving recruitment of older people to clinical trials: use of the cohort multiple randomised controlled trial design. Age and Ageing. 2015; 44: 547-550.

2. Heaven A, Brown L, Foster M, Clegg A. Keeping it credible in cohort multiple Randomised Controlled Trials: the Community Ageing Research 75+ (CARE 75+) study model of patient and public involvement and engagement. Research Involvement and Engagement. 2016; 2: 30.

#### A9 Ethical challenges for trials within cohorts (TwiCs) in low and middle income countries

##### Kate Chatfield (kchatfield@uclan.ac.uk)

###### Centre for Professional Ethics, University of Central Lancashire, Preston, UK

**Background**

Poor populations face the greatest burden from disease and disability [1] but medical research priorities are driven largely by the global market for treatments [2]. Consequently, the healthcare needs of people in low and middle income countries (LMICs) are often neglected. There is an urgent need for more pragmatic research in LMICs but clinical trials in LMICs are highly sensitive to what the European Commission describes as ‘ethics dumping’. Due to the progressive globalisation of research activities, there is a risk that research that would not be ethically permissible in the European Union is exported to countries where the legal and regulatory framework for research is not as stringent [3]. The Trials within Cohorts (TwiCs) study design offers the real world applicability of results that is desperately needed in LMICs. However, for the TwiCs design to be of value in LMICs there must be care to avoid ethics dumping with sensitivity to local preferences, needs and environment.

**Method**

A broad based consultative exercise was undertaken to identify and analyse vulnerabilities for exploitation (ethics dumping) in research in LMICs. Data was garnered from multi-level ethics bodies, policy advisors/makers, civil society organisations, funding organisations, industry and academic scholars, more than 30 members of ethics committees in LMICs, representatives from vulnerable populations in LMICs and an open call for case studies of ethics dumping in LMICs.

**Results**

A wide variety of vulnerabilities were identified and analysed thematically. For example, those who live in poor circumstances are more vulnerable to undue inducement and those who lack education may struggle to understand the research information. Cultural differences can influence the interpretation of certain ethical principles and a lack of resources and infrastructures can seriously affect the validity of the research. Additionally, many studies lack relevance for the communities in which they are undertaken and offer no potential for benefit from the results; this can leave the participants with a sense of being used or abused.

**Conclusion**

The logistical and ethical challenges for the conduct of any cohort studies in LMICs are significant. Community engagement and local ownership are essential for sustainability and the research may require significant investment in the local community. The TwiCs design offers a means of ensuring that studies are relevant to specific communities and as such, may be of great value in LMICs.

**Acknowledgements**

This investigation has arisen from a European Union’s Horizon 2020 project entitled *TRUST* (grant agreement No 664771). Thank you to members of the TRUST consortium for their input.

**References**

1. Viergever RF. The mismatch between the health research and development (R&D) that is needed and the R&D that is undertaken: an overview of the problem, the causes, and solutions. Global health action. 2013;6.

2. Evans JA, Shim J-M, Ioannidis JP. Attention to local health burden and the global disparity of health research. PLoS One. 2014;9(4):e90147.

3. Commission E. Horizon 2020, the EU framework programme for research and innovation: Ethics 2016 [Available from: https://ec.europa.eu/programmes/horizon2020/en

### Topic 3: Examples

#### A10 Experience from a prevention trial within a cohort of youth at high risk of severe mental illness

##### Rudolf Uher (uher@dal.ca)

###### Department of Psychiatry, Dalhousie University, Halifax, Nova Scotia, B3H 2E2, Canada

Severe mental illness includes schizophrenia, bipolar disorder and the most severe cases of depression. The Families Overcoming Risks and Building Opportunities for Well-being (FORBOW) program aims to find out how we can effectively prevent severe mental illness through early indicated interventions. The Trial within Cohorts (TwiCs) design makes it possible to test the long term effect of interventions with strong external validity.

FORBOW enrols children and youth (age 1-21 years) in an accelerated cohort with annual assessments of cognitive development and psychopathology [1]. Youth participants are recruited through their parents, with an oversampling of parents who are receiving health services for severe mental illness, irrespective of whether any psychopathology is present in the youth. To date, we have enrolled 317 participants in the cohort and we have been able to follow-up 95% of cohort participants annually. The combination of family history of severe mental illness and early antecedents including affective lability, anxiety, psychotic symptoms and basic symptoms allows efficient early identification of risk.

Embedded within the cohort is a trial of Skills for Wellness (SWELL), a psychological early intervention which coaches children and adolescents in coping and emotional skills needed to develop resilient mental health. Eligible participants who are 9-21 years old and present with one or more antecedents are randomly allocated to be offered the SWELL intervention or not in a 1:1 ratio. The participants are not actively seeking treatment at the time of allocation. To date, 36 participants have been randomly allocated and the intervention participation rate has been 83% with positive feedback from participating families. This contrasts with experiences from another early intervention trial in a non-help seeking population that had similar aims but followed traditional clinical trial design and was stopped because of failure to enrol participants [2,3]. The early experience suggests that the TwiCs design enables externally valid tests of preventive interventions with long-term follow up.

**Trial registration**:

ClinicalTrials.gov Identifier: NCT01980147

**References**

1. Uher R, Cumby J, MacKenzie LE, Morash-Conway J, Glover JM, Aylott A, Propper L, Abidi S, Bagnell A, Pavlova B, Hajek T, Lovas D, Pajer K, Gardner W, Levy A, Alda M: A familial risk enriched cohort as a platform for testing early interventions to prevent severe mental illness. BMC Psychiatry 2014; 14344

2. Nauta MH, Festen H, Reichart CG, Nolen WA, Stant AD, Bockting CL, van der Wee NJ, Beekman A, Doreleijers TA, Hartman CA, de Jong PJ, de Vries SO: Preventing mood and anxiety disorders in youth: a multi-centre RCT in the high risk offspring of depressed and anxious patients. BMC Psychiatry 2012; 1231

3. Festen H, Schipper K, de Vries SO, Reichart CG, Abma TA, Nauta MH: Parents’ perceptions on offspring risk and prevention of anxiety and depression: a qualitative study. BMC Psychol 2014; 2:17

#### A11 Experience from an exercise cohort multiple TwiCs Controlled Trial (cmRCT) within a hospital based breast cancer cohort: UMBRELLA FIT study

##### Anne M May^1^, Roxanne Gal^1^, Evelyn M Monninkhof^1^, Danny A. Young Afat^1,2^, Carla H van Gils^1^, Rolf HH Groenwold^1^, Helena M Verkooijen^2^

###### ^1^Department of Clinical Epidemiology, Julius Center for Health Sciences and Primary Care, University Medical Center Utrecht, Utrecht, The Netherlands; ^2^Imaging Division, University Medical Center Utrecht, Utrecht, The Netherlands

####### **Correspondence:** Anne M May (a.m.may@umcutrecht.nl)

**Introduction**

Exercise interventions show beneficial effects on cancer patients’ quality of life. However, effect sizes are often small, which might be partly explained by contamination; i.e., patients randomised to the control arm adopt the exercise intervention. Also, patients may refrain from participation, or drop-out after being randomised to the control arm. Applying a cohort multiple RCT (cmRCT) design might overcome the disadvantages of conventional RCTs when blinding of the intervention is impossible. UMBRELLA FIT studies the feasibility of cmRCT in exercise-oncology research. Effects of the intervention on quality of life will be investigated.

**Methods**

The ‘Utrecht cohort for Multiple BREast cancer intervention studies and Long-term evaluation (UMBRELLA cohort)’ started in 2013 in the UMC Utrecht (The Netherlands). Currently >1600 breast cancer patients participate. Over 85% provided broad consent to be randomly selected for future experimental interventions or to serve as control without further notice. For the UMBRELLA FIT study, 168 physically inactive breast cancer patients (12-18 months post-baseline), who gave broad consent are randomised to a 12-week supervised exercise intervention or control. Endpoints are contamination, participation, generalizability and retention (methodological) and quality of life (effectiveness). In addition, instrumental variable analysis will be performed taking drop-out/non-compliance after randomisation into account.

**Results**

The UMBRELLA FIT trial recruitment started in October 2015, since then 130 patients have been randomised. Of 65 intervention patients, 55% agreed to participate. Reasons for non-participation were mainly time constraints, dislike of exercise, or avoidance of confrontation with their disease. Acceptance rate of the intervention has been lowest in the summer period.

**Conclusion**

It is anticipated that recruitment will be completed in 2017. Results on feasibility and effectiveness will be reported.

**Trial registration**

The Netherlands National Trial Register NL.52062.041.15 / NTR5482

**Acknowledgements**

The abstract is presented on behalf of the UMBRELLA study group. We thank the study team of the UMBRELLA cohort at the department of Radiotherapy, Imaging Division and Julius Center of the University Medical Center Utrecht (UMCU) for running the cohort and collecting the data. This work was supported by a Veni grant from the The Netherlands Organisation for Health Research and Development (ZonMw 016.156.050). In addition, funding was provided by the department of Radiotherapy, Imaging Division of the University Medical Center Utrecht (UMCU), and the department of Clinical Epidemiology, Julius Center for Health Sciences and Primary Care, UMCU.

#### A12 Patient-reported outcomes in routine care: impact for TwiCs

##### Andrew Vickers (vickersa@mskcc.org)

###### Memorial Sloan Kettering Cancer Centre, New York, NY, USA

Clinicians routinely collect information on patient-reported outcomes as a part of clinical care. For instance, a rheumatologist will want to understand a patient’s level of pain and functioning in order to consider the effectiveness of a new treatment; a cancer surgeon will want to know how a patient is recovering from surgery in order to determine whether persistent symptoms require attention. There are numerous reasons why the use of standardized questionnaires for such purposes is far superior to informal discussion.

At Memorial Sloan Kettering Cancer Centre (MSKCC) we have pioneered the use of electronic methods to gather patient-reported outcomes as part of standard care. Current clinical projects include urinary and erectile function after radical prostatectomy; pain and recovery after gynaecological surgery; bowel, urinary and sexual function after rectal surgery; patient satisfaction with breast reconstruction; gerontology; pain and discomfort during prostate biopsy. We ensure that clinical staff are involved in the development of the questionnaire, the design of the report given to clinicians summarising patient responses and its integration into clinical workflow. By optimizing the clinical value of patient-reported outcomes we ensure that patients do indeed complete them in routine practice.

Data obtained to aid the clinical consultation can then be reused as the endpoints of randomised trials, facilitating the sort of clinically-integrated research associated with many TwiCs approaches. For instance, we are currently conducting a traditional randomized trial comparing two approaches to port-site closure after minimally-invasive surgery, using patient-reported hernia as an endpoint. The critical point is that all of our patients are asked to provide data on hernia, whether or not they take part in the trial. Hence, although our trial is not in a TwiCs context, it demonstrates how use of routinely collected patient-reported outcomes can facilitate the sort of low-cost, pragmatic trials common in TwiCs.

#### A13 Obtaining ethics approval for the cmRCT design from 39 ethics committees in 5 countries: the Scleroderma Patient-centered Intervention Network (SPIN)

##### Linda Kwakkenbos^1,2,3^, Marie-Eve Carrier^2^, Brett D Thombs^1,2^, and the SPIN investigators

###### ^1^Department of Psychiatry, McGill University, Montréal, Québec, Canada; ^2^Lady Davis Institute for Medical Research, Jewish General Hospital, Montréal, Québec, Canada; ^3^Behavioural Science Institute, Clinical Psychology, Radboud University, Nijmegen, the Netherlands

####### **Correspondence:** Linda Kwakkenbos (kwakkenbosl@gmail.com)

**Background**

People with rare diseases do not typically have access to evidence-based self-management and psychosocial interventions, and conducting rigorous, adequately powered trials is difficult. The Scleroderma Patient-centered Intervention Network (SPIN) is a collaboration of scleroderma centers, clinicians, patient organizations and investigators from Canada, the US, Mexico and Europe, whose aim is to develop, test, and disseminate self-management and psychosocial interventions for people living with the rare disease scleroderma [1].

**Methods**

SPIN utilizes the cohort multiple RCT (cmRCT) design to collect longitudinal data on patient-reported outcomes in scleroderma via the Internet and to test online interventions on an ongoing basis. SPIN is in the process of enrolling 2,000 scleroderma patients for an ongoing web-based cohort dedicated to better understand problems important to scleroderma patients, validating outcome measures, and informing development of interventions. SPIN will also use the cohort framework to develop, evaluate, and deliver the online support tools. Eligible participants are at least 18 years of age, have a scleroderma diagnosis, speak one of the SPIN languages (currently English, French or Spanish) and have access to the Internet. Upon enrolment in the Cohort, participants allow their physician to provide their contact information and basic medical information to the SPIN team. Once participant’s medical data are entered online, they receive emails at 3-month intervals that invite them to complete online assessments. The cmRCT design allows us to recruit very large samples for trials, even in a rare disease context, and reduces the cost of re-starting the recruitment process each time, including getting new ethics approval for each participating center.

**Results**

Since enrolment started in April 2014, SPIN has recruited over 1,500 scleroderma patients from 39 centers in Canada, the US, the UK, France and Mexico after obtaining approval from the local ethics board for each center. SPIN was recently funded to evaluate the effectiveness of an online hand exercise program and a scleroderma disease self-management program in two pragmatic RCTs embedded in the SPIN Cohort, including 400-500 patients in each. For these trials that SPIN will run through the Cohort, ethics approval is only required from the SPIN coordinating center at the Jewish General Hospital of McGill University, which adds to the feasibility of conducting multiple trials.

**Discussion**

The use of the cmRCT design and development of self-guided eHealth interventions allows SPIN to develop, rigorously test, and deliver interventions for people with a rare disease from around the world.

**Acknowledgements**

SPIN has been funded by grants from the Canadian Institutes of Health Research (TR3-119192, PJT-148504, PJT-149073) and the Arthritis Society. In addition, SPIN has received institutional contributions from the Lady Davis Institute for Medical Research of the Jewish General Hospital, Montréal, Canada and from McGill University, Montréal, Canada. SPIN has also received support from the Scleroderma Society of Ontario, the Scleroderma Society of Canada, and Sclérodermie Québec. Dr. Kwakkenbos was supported by a CIHR Banting Postdoctoral Fellowship. Dr. Thombs is supported by an Investigator Award from the Arthritis Society.

**Reference**

1. Kwakkenbos L, Jewett LR, Baron M, et al. The Scleroderma Patient-centered Intervention Network (SPIN) Cohort: Protocol for a cohort multiple randomized controlled trial (cmRCT) design to support trials of psychosocial and rehabilitation interventions in a rare disease context. *BMJ Open* 2013;3:e003563

#### A14 The ‘cohort multiple randomised controlled trial’ design for a fragile patient population

##### Joanne M. van der Velden^1^, A. Sophie Gerlich^1^, Jorrit-Jan Verlaan^2^, Helena M. Verkooijen^1,3^

###### ^1^Department of Radiation Oncology, University Medical Center Utrecht, Utrecht, The Netherlands; ^2^Department of Orthopaedic Surgery, University Medical Center Utrecht, Utrecht, The Netherlands; ^3^Julius Center for Health Sciences and Primary Care, University Medical Center Utrecht, Utrecht, The Netherlands

####### **Correspondence:** Joanne M. van der Velden (j.m.vandervelden@umcutrecht.nl)

**Introduction**

Many patients with cancer develop painful bone metastases which are a poor prognostic sign. About 60% of patients undergoing standard radiotherapy experience (partial) pain relief [1]. Stereotactic radiotherapy (SBRT) is able to deliver high-dose radiation precisely to the bone metastases and might achieve higher pain response rates [2]. In 2013, we initiated a cohort of patients with bone metastases – the PRESENT cohort – using the ‘cohort multiple Randomised Controlled Trial’ (cmRCT) design. The first randomised trial within the cohort is the VERTICAL study, comparing the effect of SBRT with standard radiotherapy in patients with metastatic bone disease [3].

**Material and Methods**

All patients with bone metastases visiting the Radiation Oncology or Orthopedic Surgery department of the UMC Utrecht are enrolled in a prospective cohort (PRESENT). Informed consent is obtained for being offered experimental interventions at random. Patients eligible for SBRT are randomised: patients allocated to the intervention group are offered the new treatment; control patients remain uninformed about the allocation. We compared inclusion rates and flow charts of the VERTICAL study with a competing classic RCT comparing SBRT with standard radiotherapy in patients with spinal metastases which started recruitment at the same time.

**Results**

Since January 2015, we have randomised 62 patients of which 27 patients were allocated to the SBRT arm (Figure). After randomisation, 6 patients were ineligible, e.g. due to too many painful lesions. Of the 21 remaining patients, 16 patients accepted SBRT. Due to rapid clinical deterioration, six patients were unable to undergo SBRT. In the competing classic RCT, 11 patients were randomised (Fig. 2) and all patients allocated to the intervention arm were able to undergo SBRT.

**Conclusion**

Comparing both trials (which are ongoing), the VERTICAL trial, using the cmRCT design, has a higher recruitment rate, and a more generalizable population as compared to the classic trial. The high drop-out rate in the intervention arm indicates that also the conduct of a cmRCT is challenging in this population.

**Trial registration**

The Netherlands Trials Register number NL49273.041.14 (PRESENT cohort) and NL49316.041.14 (VERTICAL trial). ClinicalTrials.gov registration number NCT02356497 (PRESENT cohort) and NCT02364115 (VERTICAL trial). Date of VERTICAL trial registration February 1, 2015.

**References**

1. Chow E, Zeng L, Salvo N, Dennis K, Tsao M, Lutz S. Update on the systematic review of palliative radiotherapy trials for bone metastases. Clin Oncol. 2012;24:112–24.

2. Sahgal A, Larson DA, Chang EL. Stereotactic body radiosurgery for spinal metastases: a critical review. Int J Radiat Oncol Biol Phys. 2008;71:652–65.

3. van der Velden JM, Verkooijen HM, Seravalli E, Hes J, Gerlich AS, Kasperts N, Eppinga WS, Verlaan JJ, van Vulpen M. Comparing conVEntional RadioTherapy with stereotactIC body radiotherapy in patients with spinAL metastases: study protocol for an randomised controlled trial following the cohort multiple randomised controlled trial design. BMC Cancer. 2016;16(1):909.

**Fig. 2 (abstract A14).**
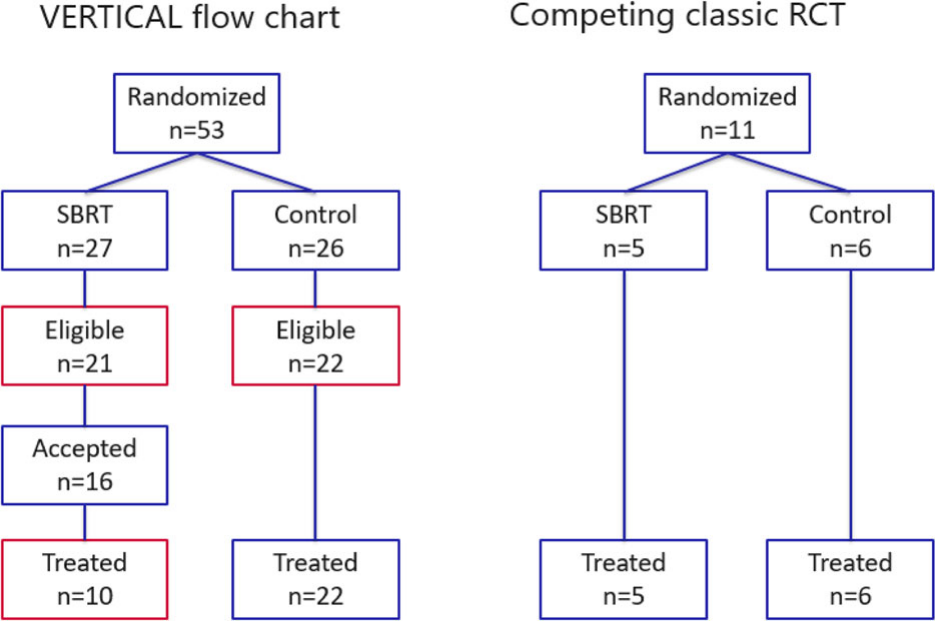
Flow charts of the VERTICAL trial and the competing classic RCT

#### A15 Multiple trials within the cmRCT design; an example within a colorectal cancer cohort

##### Alice M. Couwenberg^1^, Rolf H.H. Groenwold^2^, Rieke van der Graaf^2^, Johannes P.M. Burbach^1^, Joanne M. van der Velden^1^, Anne M. May^2^, Helena M. Verkooijen^3^

###### ^1^Department of Radiation-Oncology, University Medical Center Utrecht, Utrecht, 3508 GA, the Netherlands; ^2^Julius Center for Health Sciences and Primary Care, University Medical Center Utrecht, Utrecht, 3508 GA, the Netherlands; ^3^Imaging Division, University Medical Center Utrecht, Utrecht, 3508 GA, the Netherlands

####### **Correspondence:** Alice M. Couwenberg (a.m.couwenberg-2@umcutrecht.nl)

The cohort multiple randomized controlled trial (cmRCT)-design facilitates multiple trials in an efficient cohort structure. The design poses some methodological and ethical challenges. Here we present an example of multiple sequential trials within a cmRCT colorectal cancer cohort (PLCRC) and the challenges that were encountered.

PLCRC is a Dutch multicenter prospective cohort in which colorectal cancer patients of all stages are included. Within PLCRC, clinical data, patient reported outcome measures and biomaterials are collected. PLCRC was set up to facilitate multiple trials in a real-world setting according to the cmRCT-design. Currently, two trials are undertaken within PLCRC: the RECTAL BOOST study and the SPONGE trial. RECTAL BOOST evaluates the efficacy of boost radiation in addition to standard chemoradiation in patients with locally advanced rectal cancer [1]. SPONGE assesses the impact of the use of a retractor sponge in laparoscopic colorectal surgery on hospital stay and postoperative complication [2]. Both trials include rectal cancer patients from the same study population. Patients may therefore participate in both trials.

Ethical issues arising from multiple trials within a cmRCT-design are related to the consequences of staged-informed consent [3] and include the following: (1) Participants who have not given consent for future random selection are considered ineligible for any trial within the cohort. However, at later points in time, they may want to reconsider their eligibility for future trials. Currently, no dynamic informed consent structure within PLCRC exists; (2) Aggregated disclosure of trial results may induce disappointment of not being selected for any of the interventions in case patients were allocated to the control group (s); and (3) Broad consent for future, so called unknown studies, may not always be appropriate since some studies are known at enrollment.

Methodologically, conducting multiple trials within the same cohort may result in interacting interventions. Patients having received the boost intervention potentially have a higher risk on acute toxicity, which could result in perioperative complications and thereby prolonged hospital stay in participants of the SPONGE trial. Interacting interventions may affect the generalizability and require stratified random selection. Investigation of interactions requires substantial sample sizes. Refusal of the intervention may be related to a previous intervention, which could lead to (selection) bias and possibly impair generalizability. Also, detection of the outcome could be related to a previous intervention with hypothetically risk of differential misclassification.

Conducting multiple trials within a cmRCT cohort brings challenges, which must be taken into account when initiating a new trial.

**Trial registration** SPONGE trial Clinicaltrials.gov NCT02574013 RECTAL BOOST study Clinicaltrials.gov NCT01951521

**References**

1. Burbach JP, Verkooijen HM, Intven M, Kleijnen JP, Bosman ME, Raaymakers BW, van Grevenstein WM, Koopman M, Seravalli E, van Asselen B, Reerink O. RandomizEd controlled trial for pre-operAtive dose-escaLation BOOST in locally advanced rectal cancer (RECTAL BOOST study): study protocol for a randomized controlled trial. Trials. 2015 Feb 22;16:58.

2. Couwenberg AM, Burbach MJ, Smits AB, Van Vulpen M, Van Grevenstein WM, Noordzij PG, Verkooijen HM. The impact of retractor SPONGE-assisted laparoscopic surgery on duration of hospital stay and postoperative complications in patients with colorectal cancer (SPONGE trial): study protocol for a randomized controlled trial. Trials. 2016 Mar 10;17(1):132.

3. Young-Afat DA, Verkooijen HA, van Gils CH, van der Velden JM, Burbach JP, Elias SG, van Delden JJ, Relton C, van Vulpen M, van der Graaf R. Staged-informed Consent in the Cohort Multiple Randomized Controlled Trial Design. Epidemiology. 2016 May;27(3):389-92

#### A16 Getting past the gatekeeper: a pilot of the TwiCs design as a way of overcoming barriers to research participation in mental health studies

##### Emily Peckham^1^, Suzanne Crossland^1^, Tom Hughes^2^, Alisha O’Connor^3^, Imogen Sargent^3^ Simon Gilbody^1^

###### ^1^Mental Health and Addiction Research Group, Department of Health Science, University of York, York, UK; ^2^Leeds and York Partnership NHS Foundation Trust, Leeds, UK; ^3^Kent and Medway NHS and Social Care Partnership Trust, Kent, UK

####### **Correspondence:** Emily Peckham (emily.peckham@york.ac.uk)

**Background**

People with severe mental ill health can be a hard to reach population for trial recruitment. One reason for this is clinicians’ reluctance to invite people with severe mental ill health to take part in research. This may be due to a belief that it would not to be in the person’s best interest, without checking if this is true.

The TwiCs design might be appropriate since it is easy for participants to engage with and doesn’t cause ethical dilemmas for the recruiting clinician about whether or not it is in the participant’s best interest to take part. This promotes service user autonomy. We are piloting this design

**Methods**

We have set up the Lifestyle Health and Wellbeing Survey to ask people with severe mental ill health questions about diet, fitness, alcohol and smoking. Those who respond and are willing to be contacted again will become part of the Health and Wellbeing cohort. We are looking at people’s answers to determine whether they are potentially eligible to take part in the SCIMITAR+ randomised controlled trial (ISRCTN72955454). This trial will test an intervention to help people quit smoking. Both the Health and Wellbeing cohort and SCIMITAR+ have received research ethics committee approval. If this method of recruitment proves acceptable to participants we will embed other trials of lifestyle interventions in the Health and Wellbeing cohort.

